# Comorbidities increase in-hospital mortality in dengue patients in Brazil

**DOI:** 10.1590/0074-02760180082

**Published:** 2018-07-23

**Authors:** Guilherme L Werneck, Alejandro E Macias, Cesar Mascarenas, Laurent Coudeville, David Morley, Vincent Recamier, Mariana Guergova-Kuras, Esteban Puentes-Rosas, Nicolas Baurin, Myew-Ling Toh

**Affiliations:** 1 Universidade Federal do Rio de Janeiro, Instituto de Estudos em Saúde Coletiva, Rio de Janeiro, RJ, Brasil; 2 Universidad de Guanajuato, Departamento de Medicina, Área de Microbiología, Guanajuato, Mexico; 3 Sanofi Pasteur, Lyon, France; 4 Ariana Pharmaceuticals, Paris, France; 5 Sanofi Pasteur, Mexico

**Keywords:** dengue, haemorrhagic fever, Latin America, case fatality rate

## Abstract

Dengue remains an unmet public health burden. We determined risk factors for dengue in-hospital mortality in Brazil. Of 326,380 hospitalised dengue cases in 9-45-year-old individuals, there were 971 deaths. Risk of dying was 11-times higher in the presence of underlying common comorbidities (renal, infectious, pulmonary disease and diabetes), similar to the risk of dying from severe dengue and much higher with the combination. Ensuring access to integrated dengue preventative measures in individuals aged ≥ 9 years including those with comorbidities may help achieve the WHO objective of 50% reduction in mortality and 25% reduction in morbidity due to dengue by 2020.

In Brazil, dengue is spreading geographically with increasing numbers of reported cases and severity. Recent surveillance data indicate that between 2010-2016 Brazil suffered an average of 1,077,025 suspected, and 245,925 laboratory-confirmed dengue cases, annually (PAHO 2017). However, the true burden is likely underestimated (Sarti et al. 2016). According to Fares et al. (2015), the incidence of dengue in Brazil has frequently been high, and the number of cases in the country has at some point in time represented up to 60% of the dengue cases reported worldwide (Fares et al. 2015), with significant economic costs of US$ 728 million in 2013, mostly due to ambulatory dengue cases (Shepard et al. 2016).

The reported case fatality rate (CFR) is a measure of the severity of a disease and is defined as the proportion of reported cases of a specified disease or condition which are fatal within a specified time (WHO 2018). Dengue death rates remain high and the goal of reducing CFR for dengue haemorrhagic fever to < 1% set by the World Health Organization (WHO) and the National Dengue Control program in Brazil remains unreached (WHO 1997, MS 2002). In Brazil, the Pan American Health Organization (PAHO) has reported CFRs for dengue cases ranging from 0.04 to 4.07% annually (PAHO 2017). However, CFRs as high as 18.6% were reported for dengue patients in intensive care units (ICU) and 19.6% for in-hospital patients in a recent study in Minas Gerais state in southeastern Brazil (Amancio et al. 2015 Durand et al. 2017). As dengue CFR in Brazil may differ by two orders of magnitude from year to year, other dengue infection factors must be considered in estimating the disease severity. Comorbidities are highly prevalent in dengue endemic areas including Brazil, and contribute to some of the highest death rates and public health burden in those countries (Mehta and Hotez 2016). Dengue patients with comorbidities may be at higher risk of severe dengue and death, however, there have been no large scale studies conducted in Brazil (Figueiredo et al. 2010 Teixeira et al. 2015 Toledo et al. 2016).

In a large retrospective cohort study, we determined risk factors for dengue in-hospital mortality using the Brazilian Hospital Information System of the Unified Health System (SIH/SUS) database over an eight year period (2008-2015) (Ministério da Saúde - Available from: http://sihd.datasus.gov.br/). Hospital admission records (92 million) from 20,576 hospital departments (e.g. pediatric, clinical) at 5,983 hospitals were included. Patient records from 326,380 hospitalised dengue cases in 9-45-year-old individuals [173,778 female (53.2%) and 152,602 male (46.8%)] were analysed. This broad age range includes adolescents, young adults, and adults, thereby excluding the elderly who may have a high prevalence of comorbidities and thus limit the ability to detect differences in CFR. Ethical approval was not required as the database was publically accessible. In-hospital mortality was identified with dengue cases and comorbidities identified directly from the recorded principal and secondary diagnoses for each hospitalised dengue case using the International Classification of Diseases, 10th Revision (ICD 10) codes (WHO 2014). Dengue cases were divided into either non-severe (ICD 10 code = A90; classical dengue) or severe dengue diagnosis codes (ICD 10 code = A91; dengue haemorrhagic fever).

In order to exclude potential bias in the analysis, as comorbidities may also be a consequence of severe dengue manifestations, codes considered as severe dengue symptoms and complications were excluded. The comorbidity definitions used are summarised in Supplementary data, Table. Analyses were performed using the KNIME (Berthold et al. 2008) analytic platform integrated with KEM^®^ (Ariana Pharmaceuticals; Liquiere and Sallantin 1998) data mining tools, MySQL database and R v3.0.3 statistical software. Data were presented as CFRs and relative mortality ratios (RMR) and 95% confidence intervals (CI) compared to different groups. P values for between group RMRs were determined for statistical significance by Fisher’s exact test, whereby p < 0.05 was considered statistically significant.

We first determined whether mortality rates were increased in hospitalised dengue patients with underlying comorbidities compared to those without comorbidities. Of the 326,380 hospitalised dengue cases in 9-45-year-old individuals there were 971 deaths resulting in an overall CFR of 0.30% in this age group (Table). The crude RMR from hospitalised dengue was 11-times higher (95% CI 9-15) in the presence of common comorbidities (CFR 3.07%, p < 0.001) compared to the absence of comorbidities (CFR 0.27%).

**TABLE:** Summary of comorbidities recorded in hospitalised dengue cases aged 9-45 years at admission (Brazilian Hospital Information System of the Unified Health System (SIH/SUS), 2008-2015)

Group	Cases	Female	All dengue diagnoses (A90, A91)				Dengue fever diagnosis (A90)				Dengue haemorrhagic fever diagnosis (A91)				
		
			Male	Deaths	CFR (%)	RMR[1] (95% CI)	Cases	Deaths	CFR (%)	RMR[2] (95% CI)	Cases	Deaths	CFR (%)	RMR[2] (95% CI)	Prevalence[3] (%)
All dengue cases	326,380	173,778	152,602	971	0.30		310,387	501	0.16		15,993	470	2.94		4.9
with no 2nd diagnosis	320,793	170,726	150,067	862	0.27	1	305,311	440	0.14	1	15,482	422	2.73	19 (17-22)***	4.8
with comorbidity	1629	871	758	50	3.07	11 (9-15)***	1492	29	1.94	13 (9-20)***	137	21	15.33	106 (71-159)***	8.4
infectious diseases	657	309	348	25	3.81	14 (10-21)***	609	15	2.46	17 (10-28)***	48	10	20.83	145 (83-253)***	7.3
pulmonary disorders	364	192	172	13	3.57	13 (8-23)***	330	6	1.82	13 (6-28)***	34	7	20.59	143 (73-278)***	9.3
urinary disorders	258	180	78	1	0.39	1 (0.2-10) ns	251	1	0.40	3 (0.4-20) ns	7				2.7
renal disease or failure	82	37	45	6	7.32	27 (13-59)***	69	4	5.80	40 (15-105)***	13	2	15.38	107 (30-383)***	15.9
HIV	70	33	37	1	1.43	5 (0.8-37) ns	59	1	1.69	12 (2-82) ns	11				15.7
diabetes	61	37	24	2	3.28	12 (3-48)*	57	1	1.75	12 (2-85) ns	4	1	25.00	173 (32-950) **	6.6
heart failure	8	3	5	1			5	1			3				
obesity	7	4	3				5				2				
stroke	3	1	2	1			2				1	1			
ischemic heart disease	1		1				1								

CFR: case fatality rate; RMR[1]: relative mortality rate calculated relative to CFR for all dengue cases with no 2nd diagnosis; RMR[2]: relative mortality rate calculated relative to CFR for dengue cases with A90 diagnosis and no 2nd diagnosis; *: p < 0.05; **: p < 0.01; ***: p < 0.001; ns: p > 0.05; [3] Prevalence: percentage of dengue cases with dengue haemorrhagic fever diagnosis (A91); *ǂ*: infectious diseases: dengue cases with a secondary (comorbidity) diagnosis of “infectious diseases” (excluding dengue) recorded; i.e. patient had dengue and another infectious disease (ICD10 A code).

The risk of dying from hospitalised dengue in those with non-severe dengue and underlying comorbidities (CFR 1.94%, RMR 13, 95% CI 9-20, p < 0.001) was not statistically different to the risk of dying from severe dengue haemorrhagic fever with no underlying comorbidity (CFR 2.73%, RMR 19, 95% CI 17-22, p < 0.001) (Table). The risk of dying was further significantly increased with the combination of severe dengue haemorrhagic fever and underlying comorbidities (CFR 15.33%, RMR 106, 95% CI 71-159, p < 0.001) compared to individuals with dengue alone (non-severe dengue and no underlying comorbidity). Furthermore, there was a 1.7-fold higher prevalence of severe dengue associated with comorbidities (8.4%, p < 0.001), compared to without comorbidities (4.8%) (Table).

Thus, the presence of both comorbidities and severe dengue are cumulative risk factors for increased hospitalised dengue death rates. Comorbidities with highest dengue hospital death rates, regardless of dengue severity, were consistently renal disease, infectious disease, pulmonary disease and diabetes. However, in the presence of both severe dengue and underlying comorbidities, such as diabetes (CFR 25%), other non-dengue infectious diseases (CFR 20.83%), pulmonary disorders (CFR 20.59%), renal disease (CFR 15.38%); fatality rates were higher than in individuals with non-severe dengue and no underlying comorbidities (Table).

To our knowledge, this is the largest retrospective cohort study of more than 300,000 hospitalised dengue cases (15,993 dengue haemorrhagic fever, 971 fatal cases) demonstrating a high mortality in the presence of comorbidities in individuals 9-45 years of age in Brazil over an eight year period. Our study demonstrated for the first time that risk of dengue death in the presence of comorbidities is similar to the risk of death from severe dengue and much higher with the combination of both comorbidities and severe dengue. The comorbidities contributing to increased death rates were consistently infectious diseases, pulmonary diseases, renal disease/failure and diabetes regardless of dengue severity.

Prior publications on the impact of underlying comorbidities on dengue related mortality are limited and inconsistent, in part related to the small number of deaths included in these analyses (Toledo et al. 2016). However, these reports suggest that underlying comorbidities can worsen dengue clinical outcomes.

Two large scale studies explored severe dengue as a risk factor for dengue mortality but did not specifically analyse the contribution of comorbidities. Both studies used the Brazilian Information System for Notifiable Diseases (SINAN) and gathered information on 281,159 and 105,459 dengue cases and 156 and 62 deaths, respectively (Campos et al. 2015, Pinto et al. 2016). Campos et al. (2015) reported that factors associated with dengue mortality included age > 65 years and plasma leakage. Pinto et al. (2016) found that patients who died due to severe dengue had more hematuria, gastrointestinal bleeding, and thrombocytopenia than survivors. In Brazil, there have been two further publications on a small series of patients. In a case-controlled study with 1345 patients during 2003-2005 (n = 170 dengue haemorrhagic fever cases), diabetes (OR 2.75; 95% CI 1.12-6.73) was associated with dengue haemorrhagic fever (Figueiredo et al. 2010). In a later case-controlled study with 1806 patients during 2009-2012, hypertension (OR 1.6; 95% CI 1.1-2.1) and skin allergy (OR 1.8; 95% CI 1.1-3.2) increased the likelihood of progressing to dengue haemorrhagic fever but diabetes was not a confirmed risk factor (Teixeira et al. 2015). A study of the 1981 dengue epidemic in Cuba found that chronic diseases such as bronchial asthma, diabetes mellitus and sickle cell anaemia were more frequently observed in fatal dengue cases (Bravo et al. 1987). A number of other studies have also suggested that underlying comorbid conditions are common among dengue-related deaths, but these have generally been based on analyses that included very few deaths (typically, < 15 deaths included in the analyses) (Guzman et al. 1999, Lee et al. 2005, Ong et al. 2007, Lahiri et al. 2008, Lee et al. 2008). The estimated CFR of 0.3% in all hospitalised dengue cases in our study was much lower than the 1.3% average reported to PAHO for Brazil during the same time period, but this may be due to differences in reporting of severe dengue, which may elevate the CFR in other studies. Furthermore, this may reflect the inherent differences in clinical case management practices and dengue diagnosis for dengue/severe dengue (Kalayanarooj 1999), or differences in circulating dengue virus serotypes, which all co-circulate but vary unpredictably over time (Ocazionez et al. 2006, OhAinle et al. 2011). In our study, although dengue diagnosis was based on a combination of clinical diagnosis and/or laboratory diagnosis, this reflects current clinical practice guidelines by the WHO in countries where laboratory diagnosis may not be available. We have also conducted a more in-depth analysis of the individual risk factors and determined that age, severe dengue and comorbidities are independent and cumulative risk factors for increased dengue mortality in Brazil and other countries in Latin America (submitted for publication). Despite the fact that we excluded all severe dengue symptoms and pathologies associated with severe dengue outcome, it is possible that there remains still some underlying bias whereby increased risk of death from comorbidities may be just a manifestation of severe dengue induced comorbidities. For example, we considered renal disease or failure as a comorbid condition, but acute renal failure may be a complication of severe dengue (Wiwanitkit 2005). However, overall, the data on renal disease also included chronic renal failure and other underlying renal pathologies which are not outcomes of severe dengue. Socioeconomic and healthcare factors may also play a role in dengue mortality; in Brazil, dengue mortality was shown to be associated with a number of socioeconomic and urbanisation indicators (Paixão et al. 2015). Nonetheless, a major strength of our study is the large number of deaths assessed in our analysis, which encompasses reported cases across the whole of Brazil. Further, our study used the SIH/SUS reporting system which is unique in allowing the analysis of comorbidities in a detailed way.

In conclusion, the risk of dying from hospitalised dengue is 11-times higher in those with underlying common comorbidities. To our knowledge this is the largest retrospective analysis conducted in Brazil. The comorbidities identified in a high proportion of dengue deaths were renal disease, infectious disease, pulmonary disease and diabetes. Ensuring access to dengue preventative measures (e.g., dengue vaccine, vector control and prompt medical care) in individuals with comorbidities may help achieve the WHO objective of 50% reduction in dengue-related mortality and 25% reduction in morbidity by 2020 (WHO 2012).
