# Supplementary material for: Comorbidities increase in-hospital mortality in dengue patients in Brazil
Source: Mem Inst Oswaldo Cruz. 2018 Jul 23;113(8):e180082. doi: 10.1590/0074-02760180082 (PMC6056917; doi:10.1590/0074-02760180082)
Supplement: Supplementary file 1 [file 0074-0276-mioc-113-08-e180082-Suppl01.pdf]

TABLE

ICD10 code definitions for comorbidity groups

Comorbidity	ICD10 Code	Description
Diabetes	E10	Insulin-dependent diabetes mellitus
	E11	Non-insulin-dependent diabetes mellitus
	E13	Other specified diabetes mellitus
	E14	Unspecified diabetes mellitus
HIV	B20	Human immunodeficiency virus (HIV) disease resulting in infectious and parasitic diseases
	B22	HIV disease resulting in other specified diseases
	B23	HIV disease resulting in other conditions
	B24	Unspecified HIV disease
Heart failure	I50	Heart failure
Ischemic heart disease	I20	Angina pectoris
	I21	Acute myocardial infarction
	I24	Other acute ischemic heart diseases
	I25	Chronic ischemic heart disease
Obesity	E66	Obesity
Pulmonary disorders	J12	Viral pneumonia, not elsewhere classified
	J15	Bacterial pneumonia, not elsewhere classified
	J18	Pneumonia, organism unspecified
	J21	Acute bronchiolitis
	J40	Bronchitis, not specified as acute or chronic
	J44	Other chronic obstructive pulmonary disease
	J45	Asthma
	J90	Pleural effusion, not elsewhere classified
	J96	Respiratory failure, not elsewhere classified
Renal disease or failure	N10	Acute tubulo-interstitial nephritis
	N11	Chronic tubulo-interstitial nephritis
	N12	Tubulo-interstitial nephritis, not specified as acute or chronic
	N13	Obstructive and reflux uropathy
	N18	Chronic kidney disease
	N17	Acute renal failure
	N19	Unspecified kidney failure
Stroke	I64	Stroke, not specified as haemorrhage or infarction
Urinary disorders	N39	Other disorders of urinary system
Infectious disease	A00-A99	Infectious diseases (excluding dengue A90/91)

CC BY
